# Anatomic reconstruction for major tissue loss following abdominoplasty: A case report

**DOI:** 10.1016/j.ijscr.2020.06.019

**Published:** 2020-06-11

**Authors:** Vladislav Pavlovich Zhitny, Noama Iftekhar, Barry Zide, Frank Stile

**Affiliations:** a School of Medicine, University of Nevada, Las Vegas, Las Vegas, NV, USA; b School of Medicine, Loyola University of Chicago, Maywood, IL, USA; c New York University, Langone Health, New York City, NY, USA; d Stile Aesthetics, Las Vegas, NV, USA

**Keywords:** Abdominoplasty, Skin flap necrosis, Plastic surgery, Cosmetic surgery, Tummy tuck, Case report

## Abstract

**Introduction:**

Abdominoplasty is currently one of the most widely requested cosmetic procedures in the United States. There is limited literature describing the corrective measures following skin loss from a flap necrosis due to an abdominoplasty complication.

**Methods:**

A 53-year-old female presented with a large split thickness skin graft due to skin loss as a necrotic complication from an abdominoplasty performed at our center. The patient suffered from emotional stress related to the abdominal deformity, which included a lack of an umbilicus.

**Results:**

Patient recovered well post-operatively. She required two procedures – the initial procedure involved excision of the skin graft and closure with the patient’s own full-thickness skin and tissue. The second procedure, which occurred six months later, involved the creation of an umbilical stalk from a small cuff of skin.

**Conclusion:**

Abdominoplasty, although sought for cosmetic purposes, is indeed a major procedure and can result in complications especially from aggressive liposculpture. When complications do occur, revisions are indeed possible with aesthetically pleasing results.

## Highlights

• Abdominoplasty is a highly requested cosmetic procedure, but ultimately, as a major surgery can have unwanted complications.
• Autografts are a viable option for repairing skin necrosis, especially after a tight liposculpture.
• Large skin grafts may result in abdominal wall deformity, which requires surgical creativity for repair.
• Abdominal wall deformity can be corrected in a two-part procedure.

## 1 Introduction

First described in the literature in 1899, abdominoplasty, also commonly known as “tummy tuck,” is a procedure intended for revision of excessive abdominal skin and fat as well as strengthening of abdominal wall [1]. It is the fifth most common plastic surgical procedure performed in the United States with over 130,081 tummy-tucks performed in 2018 alone [2]. Abdominoplasty is most frequently requested by women, especially those with significant weight gain and loss, post-pregnancy associated cosmesis issues, ambulation difficulties, and urinary incontinence [[3], [4], [5]]. The procedure has a high patient satisfaction rate with associated improved self-image.

Infra-umbilical redundant skin and fat are excised. In some patients, this may pose its own set of risks. Complications can range from post-surgical hematomas, seromas or infections. Skin flap necrosis, a complication of this procedure, is a rare sequalae that can occur due to interrupted perfusion, anatomic aberrations of the vasculature, poor flap design, pathology associated with lupus or diabetes, smoking or use of tight garments post-surgery [[6], [7], [8], [9]]. Aggressive liposculpture of the central abdomen performed concurrently may also increase the risk of flap loss. The different types of abdominoplasty include a mini-abdominoplasty (removal of excess skin and fat in the naval area), modified abdominoplasty (removal of excess fat and skin from the abdomen with adjustment of the umbilicus position and strengthening of skin and muscle), or the full abdominoplasty (which includes removal and strengthening of the skin, fat, and muscle in areas not limited to the lower abdomen) [10].

There is limited literature discussing corrective cosmetic measures that may be employed following skin loss secondary to flap necrosis following abdominoplasty. Here we describe a case where a multi-layer anatomic approach was used to excise a previously placed skin graft and repair the defect that resulted from significant flap loss following elective cosmetic abdominoplasty. This case has been reported in line with SCARCE criteria [11].

## 2 Case-presentation

**Fig. 1:**
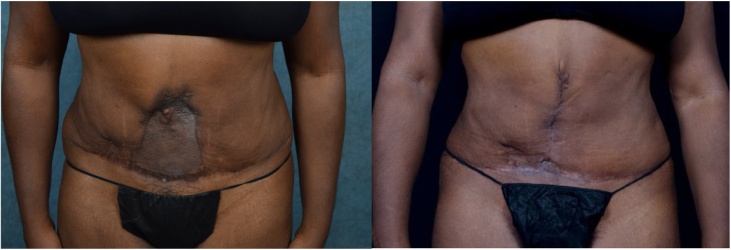
Left: Split-thickness skin graft resurfacing of abdominal defect (ventral view). Right: Status post wound excision and closure.

**Fig. 2:**
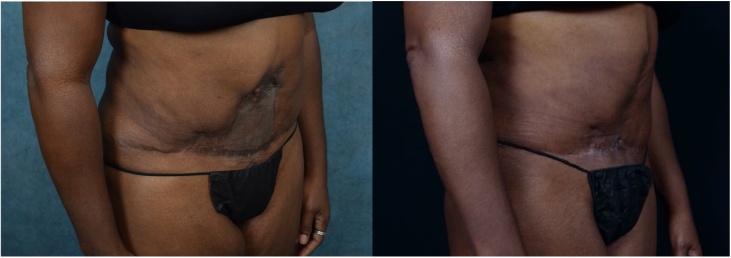
Left: Split-thickness skin graft resurfacing of abdominal defect (oblique view). Right: Status post wound excision and closure.

A 53-year-old African American female presented to the clinic for evaluation. She had a large abdominal deformity that resulted from significant skin loss following elective abdominoplasty. A split thickness meshed skin graft was used to cover the defect after the original procedure (Fig. 1, Fig. 2). Patient’s experience and resulting abdominal deformity caused her to have a significant emotional distress, depression and a distorted self-image. This deformity was further complicated by the loss of the patient’s umbilicus.

Her past medical history included a diagnosis of a monitored pituitary microadenoma, obesity and type 2 diabetes mellitus. Her surgical history was significant for abdominoplasty and mastopexy that were performed two years prior to our initial encounter. She had a split-thickness skin graft placed skin on the abdomen for resurfacing of her abdominal soft tissue defect. The only medication this patient was actively taking was metformin.

Prior to the surgical procedure, the patient weighed 81.64 kg and her height was 1.67 m (Body mass index: 29.1 kg/m^2^). Patient’s preoperative laboratory and radiology reports were normal. Physical examination did not present us with any obstacles to our reconstruction. Patient was observed to be mildly hypertensive with a blood pressure of 133/98. The patient was deemed to be in good health and cleared for her revision surgery.

## 3 Operative procedure

The abdomen is divided into three zones. Zone 1 is an area extending from Xiphoid process superiorly to the area just below the umbilicus inferiorly and is bound laterally by the lateral edges of the rectus muscle. The blood supply to zone 1 is derived from the superficial branches of the superior and inferior epigastric vessels. Zone 2 is the area below a line drawn between the iliac spines. It derives its blood supply from the circumflex iliac and external pudendal vessels. Zone 3 is the skin lateral to the lateral edge of the rectus muscle and posterior bilaterally. Zone 3 blood supply is derived from the posterior and lateral perforating branches of the intercostal, subcostal and lumbar vessels.

The central area comprised of Zone 1 and 2 may be considered a “water-shed” area when the abdominal skin and fat flap is elevated off the rectus and oblique muscular fascia. This makes these two zones more susceptible to vascular compromise.

The intent of this patient’s initial procedure was to excise her meshed skin graft and close the resulting defect with her own full thickness skin and soft tissue. In the operating room and under general anesthesia, we began by de-epithelializing the central meshed skin graft. The wound measured approximately 13 by 21 cm in its greatest dimensions. A small cuff of skin, which would be used later to create the new umbilical stalk and umbilicus, was preserved. A margin of skin was excited from the wound’s edge about 0.5 cm in width.

Next, beginning centrally and proceeding laterally and superiorly, full-thickness skin and fat flaps were elevated with the abdominal wall fascia as the limit for deep dissection. These flaps extended to anterior axillary lines bilaterally, and to the xiphoid and costal margins superiorly.

A 2−0 double looped nurolon suture was employed to correct the diastasis recti, which was not repaired during her initial procedure. This suture extended from the xiphoid to the pubis. Correction off this patient’s diastasis assisted in reducing the cross-sectional area off her abdominal defect.

Next, the skin and fat flaps were advanced and approximated in the midline under modest tension. We were comfortable with this because the flaps were already delayed from her previous abdominal surgery.

Jackson-Pratt closed suction drains were placed and allowed to exit the lateral aspect of the lower abdominal wound bilaterally.

## 4 Results-follow-up

This patient did well post-operatively. Drains were removed at seven and fourteen days following this procedure. The wound remained intact and she resumed full activity four weeks post-operatively.

**Fig. 3:**
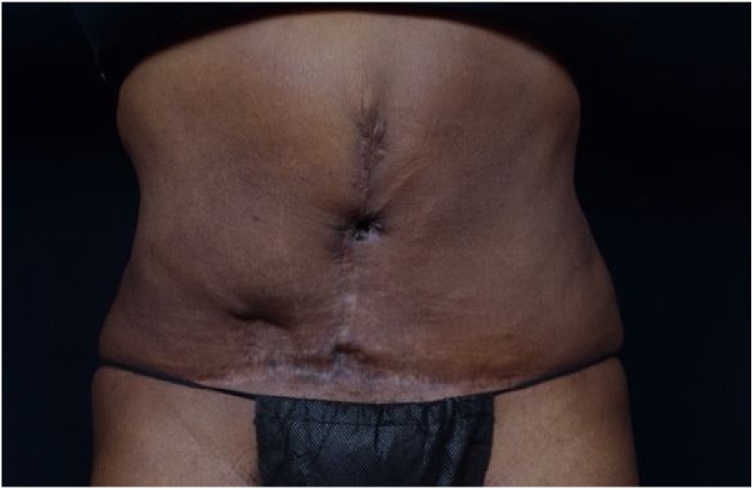
Creation of umbilicus following correction of abdominal defect.

She returned to the operating room several months later (Fig. 2). The previously created umbilical stalk was brought out through a small opening in her vertical abdominal scar (Fig. 3). Since her last surgery, this patient has not had any new wound issues and is happy with her result. The patient was offered a fat graft procedure to smooth out the contour but she elected to forgo this procedure at this time.

## 5 Discussion

Classified as a major surgery, abdominoplasties do carry a certain degree of risk. While this plastic surgery technique can help improve a patient’s abdominal appearance, temporary numbness and seromas are very commonly encountered. Skin necrosis is a rare complication of abdominoplasty but causes significant challenges for both the patient and surgeon when it does occur.

In small cases of tissue loss, aggressive and focused wound care protocols can expedite debridement and wound closure without the need for revisional surgery. Larger wounds require debridement for removal of the non-viable soft tissue, wound care and either local tissue advancement or rearrangement for satisfactory closure. When the area of necrosis is very large, such as in our patient, this may initially require operative debridement and placement of a skin graft in anticipation of future procedures. Skin grafts are often useful in patients with a history of poor wound healing.

Post-operative complications are especially onerous for the surgeon and distressing to patients when they occur following elective cosmetic procedures. Patients elect to have cosmetic surgery with the intent of achieving an enhanced appearance. Poor aesthetic results following complications from abdominoplasty are less than ideal.

While recent advancements in techniques and post-operative monitoring have improved outcomes, the vascularity of the newly created flap is not always predictable. These challenges related to perfusion can cause significant tissue loss. This patient’s chronic diabetes may have also unfortunately predisposed her to this poor outcome [12].

Tissue expanders and repeated skin advancement are frequently required if skin necessary laxity will not allow for tension free wound coverage [13]. The skin and soft tissue bordering this patient’s abdominal defect was judged to have sufficient laxity, enough to advance our flaps and cover her defect. In future grafts, tissue expanders and repeated skin advancement may be preventative to the surgical procedures outlined above. We were able to approximate the edges of normal viable skin without undue tension following de-epithelialization of the meshed skin graft. This created an aesthetic outcome that would otherwise be remote. Nearly 21 % of patients having an abdominoplasty procedure require revision surgeries [14]. While skin complications, including necrosis, do occur in abdominoplasties, we were unable to locate literature that discussed outcomes or techniques such as skin grafts or their revisions. Skin complications post-abdominoplasty are rare. Scar removal typically follows with shave excision or a small incision around the scar incision with re-approximation. Skin graft and tissue excision are rare and mostly used on large scars. This usually creates a natural blend in the rare instances when it is used, but in our patient’s case the skin graft was used to repair a large area of necrosis. It is rare to see such a large deformity in the few scar revisions that occur. Because of this, two procedures rather than one procedure were required for repair.

## 6 Conclusion

Abdominoplasty is a versatile procedure but is often associated with a high rate of complications. When complications do occur, this procedure can be successfully revised to correct these producing more aesthetically pleasing outcomes.

## Declaration of Competing Interest

No disclosures.

## Ethical approval

Approval exempt as this is a case study and involves less than 2 patients. Appropriate media consent forms obtained from patient.

## Consent

This is a case study. All relevant characteristics that could be identifiable have been removed or changed. Written informed consent was obtained from the patient for publication of this case report and accompanying images. A copy of the written consent is available for review by the Editor-in-Chief of this journal on request.

## Author contribution

Vladislav Zhitny came up with the concept. Vladislav Zhitny and Noama Iftekhar evenly contributed to the literature search and wrote the article. Barry Zide conducted the final review of the document. Frank Stile is the operating surgeon and conducted final review of the document.

## Registration of research studies

N/A as this is not a study.

## Guarantor

Dr. Frank L Stile.

## Provenance and peer review

Not commissioned, externally peer-reviewed.
